# *Anaplasma phagocytophilum* Ats-1 enhances exosome secretion through Syntenin-1

**DOI:** 10.1186/s12866-023-03023-4

**Published:** 2023-09-27

**Authors:** Ruirui Li, Zhongchen Ma, Wei Zheng, Yangyang Xiao, Zhen Wang, Jihai Yi, Yong Wang, Chuangfu Chen

**Affiliations:** 1 International Research Center for Animal Health Breeding, College of Animal Science and Technology, Shihezi University, Shihezi, China; 2 Collaborative Innovation Center for Prevention and Control of High Incidence Zoonotic Infectious Diseases in Western China, College of Animal Science and Technology, Shihezi University, Shihezi, China; 3 Present Address: Shihezi University, Shihezi City, Xinjiang Uygur Autonomous Region China

**Keywords:** *A. phagocytophilum*, Effector protein Ats-1, Syntenin-1 (SDCBP), Exosome

## Abstract

*Anaplasma phagocytophilum* is an intracellular obligate parasite that causes granulocytic anaplasmosis. Effector Ats-1 is an important virulence factor of *A. phagocytophilum*. Multiomics screening and validation has been used to determine that Ats-1 regulates host cell apoptosis and energy metabolism through the respiratory chain mPTP axis. In this study, a total of 19 potential binding proteins of Ats-1 in host cells were preliminarily screened using a yeast two-hybrid assay, and the interaction between syntenin-1 (SDCBP) and Ats-1 was identified through immunoprecipitation. Bioinformatics analysis showed that SDCBP interacted with SDC1, SDC2, and SDC4 and participated in the host exosome secretion pathway. Further studies confirmed that Ats-1 induced the expression of SDC1, SDC2, and SDC4 in HEK293T cells through SDCBP and increased the exosome secretion of these cells. This indicated that SDCBP played an important role in Ats-1 regulating the exosome secretion of the host cells. These findings expand our understanding of the intracellular regulatory mechanism of *A. phagocytophilum*, which may enhance its own infection and proliferation by regulating host exosome pathways.

**Supplementary Information:**

The online version contains supplementary material available at 10.1186/s12866-023-03023-4.

## Introduction

Granulocytic anaplasmosis is a tick-borne disease caused by *Anaplasma phagocytophilum*. *Anaplasma phagocytophilum* infects the granulocytes and endothelial cells in ruminants, horses, dogs, and humans [1–4]. After the animal is infected, clinical manifestations such as fever, discomfort, headache, arthralgia, nausea, vomiting, and cough often occur [2, 5, 6]. *Anaplasma phagocytophilum* is spreading in North America, East Asia, and Eurasia, and their geographic location and overall incidence are increasing [7–12]. Preventing and controlling this disease is difficult.

*Anaplasma phagocytophilum* can adapt to, escape from, or resist various mechanisms of killing (or inhibiting) bacteria in the host cell (the neutrophils). It accomplishes this by forming inclusion bodies in the host cell, inhibiting host cell apoptosis or inducing autophagy, and downregulating the inflammatory response in order to survive and reproduce [4, 13, 14]. Studies have shown that after infecting host cells, *A. phagocytophilum* can secrete a variety of effector proteins that act on various signaling pathways to regulate cell differentiation and proliferation, the immune response, lysosomal fusion, apoptosis, and signal transduction [15, 16]. However, the molecular mechanisms by which *A. phagocytophilum* regulates host cells remain unclear and need to be further studied.

In *Brucella* and *Legionella pneumophila*, the Type IV secretion system (T4SS) is encoded by many genes that transfer DNA or proteins to eukaryotic cells and change the key functions of the host cells, thereby facilitating bacterial reproduction and survival [17–19]. A gene encoding the T4SS exists in *A. phagocytophilum* [20]. To date, several T4SS effector proteins in *A. phagocytophilum* have been identified, such as Ankyrin repeat domain-containing protein A (AnkA), *A. phagocytophilum* toxin A (AptA), and *Anaplasma* translocated substrate 1 (Ats-1). The secreted AnkA is located in the cytoplasm and nucleus of host cells, and it can activate SHP-1 in addition to binding to Abi-1 and the main tyrosine phosphorylation proteins in infected cells [21]. AptA mediates ERK1/2 activation and interacts with the intermediate fibrin vimentin to promote the survival of *A. phagocytophilum* in the host cell [22].

It has been reported that *A. phagocytophilum* Ats-1 protein, as a virulence factor of the pathogen, plays an important role in regulating pathogen invasion, intracellular survival and reproduction, and the biological processes of host cells [23]. Some Ats-1 proteins are localized in the mitochondria of infected cells, inducing anti-apoptosis and energy metabolism by upregulating the respiratory chain-mPTP axis in eukaryotic mitochondria [24]. Most Ats-1 proteins are located in the host cytoplasm and bind Beclin 1 to induce autophagy in host cells [14]. The detailed signaling mechanism and pathways of this protein need to be further studied to determine whether this protein is involved in regulating other biological processes in host cells [23].

Exosomes are polyvesicular bodies released intracellularly that are characterized by a diameter between 30 and 150 nm (with an average of approximately 100 nm), as well as a distinctive cup-shaped membrane structure [25]. Exosomes are produced both under normal physiological conditions and in response to external environmental stimuli. Exosomes contain membrane proteins, cytoplasmic nuclear proteins, extracellular matrix proteins, metabolites, and nucleic acids (including mRNA, non-coding RNA, and DNA). Exosomes can exert autocrine, paracrine, and endocrine functions [26], and the bioactive molecules present in them are closely related to the type and physiological and pathological state of the donor cells. The contents of exosomes can still maintain their biological functions when they are delivered to recipient cells, promote phenotypic changes in recipient cells, and affect their physiological state [27].

In addition to apoptosis and autophagy, the Ats-1 protein may also regulate other functions of host cells, though its specific molecular mechanisms remain unclear. Moreover, it has been reported that HEK293T cells are susceptible to *A. phagocytophilum* infection [28, 29]. We screened the Ats-1 interaction protein, syndecan-binding protein (Syntenin-1 [SDCBP], NC_000008), from human HEK293T cells using the yeast two-hybrid technique. Ats-1 induced the expression of SDC1, SDC2, and SDC4 in HEK293T cells through SDCBP and increased the exosome secretion of HEK293T cells. These results provide a foundation for studying the biological functions of Ats-1 in the host cytoplasm.

## Materials and methods

### Strains, cells, and reagents

*Escherichia coli* DH5α was provided by the Collaborative Innovation Center for the Prevention and Control of Infectious Diseases in Western China. HEK293T cells were purchased from the Cell Resource Center at the Institute of Basic Medical Sciences of the Chinese Academy of Medical Sciences/Peking Union Medical College (Beijing, China) and cultured using Dulbecco’s Modified Eagle Medium (DMEM) (Solarbio, Beijing, China) containing 10% fetal bovine serum (FBS) (Gibco, Waltham, MA, USA) under 5% CO_2_. The *Y*_*2*_*H* gold (*MATα*) strain (Clontech, Mountain View, CA, USA) was incubated with YPD Plus (Coolaber, Beijing, China), Dosupplement-Leu/-Trp (Coolaber, Beijing, China), and Dosupplement-Ade/-His/-Leu/-Trp (Coolaber, Beijing, China). The exosome extraction and purification kit (cell supernatant), exosome extraction and purification kit (other body fluids), and exosome protein lysate used were all purchased from Umibio (Shanghai, China). The BCA protein quantitation kit was purchased from Sigma Aldrich (St. Louis, MO, USA).

### Construction of the vector and transfection model

The total RNA of HEK293T cells was extracted using an RNeasy Mini Kit (Qiagen, Mississauga, ON, Canada), and cDNA synthesis and purification were carried out using a Make Your Own “MATE & PLATE” Library System (Clontech, Mountain View, CA, USA) that was linked to the carrier pGADT7 (Clontech, Mountain View, CA, USA).

The Ats-1 gene was synthesized by Sangon Biotech (Shanghai, China) based on the sequence of the *A. phagocytophilum* Ats-1 gene (FJ210653) in the Gene Database (https://www.ncbi.nlm.nih.gov/). The Ats-1 gene was ligated into a pGBKT7 (Clontech, Mountain View, CA, USA) vector to construct a pGBKT7-Ats-1 recombinant vector. The Ats-1 gene was fused with a His tag and connected to a pcDNA 3.1 (Invitrogen, Carlsbad, CA, USA) vector to construct a pcDNA3.1-Ats-1 recombinant vector. The recombinant plasmid pcDNA3.1-Ats-1 was extracted using a Large-scale Plasmid Extraction Kit (Tiangen, Beijing, China) for transfection.

The SDCBP (Gene ID: Mature 6386) transcript was searched in the NCBI database, and siRNA SDCBP was designed using the Invitrogen online website (https://rnaidesigner.thermofisher.com/rnaiexpress/blast.jsp). Three siRNA SDCBPs were designed and synthesized, and their specific sequences are shown in Table S3. The HEK293T cells were cultured to a density of 70–80%, and either the recombinant plasmid pcDNA3.1-Ats-1 or siRNA SDCBP was transfected into HEK293T cells using Lipofectamine 3000 Transfer Reagent (ThermoFisher, Waltham, MA, USA). After 4–6 h, the cultures were continued in DMEM containing 10% FBS at 37 °C in a 5% CO_2_ incubator.

### Expression of Ats-1 protein in HEK293T cells verified by western blotting

Immunoblotting analysis of the Ats-1 protein was performed through SDS-PAGE (CWBIO, Beijing, China) using mouse anti-6 × His-tag antibody (Abcam, Cambridge, MA, USA) (1:1000) as the primary antibody. Goat Anti-mouse IgG H&L (Abcam, Cambridge, MA, USA) (1:3000) was implemented as the secondary antibody, and a SuperSignal West Femto Trial Kit (ThermoFisher, Waltham, MA, USA) was utilized for color development. Actin was utilized as a control. Mouse Anti-β-actin Monoclonal Antibody (Sino Biological, Beijing, China) (1:1000) was used as the primary antibody and Goat Anti-Mouse IgG H&L (Abcam, Cambridge, MA, USA) (1:3000) was used as the secondary antibody in our research. And the blots were cut prior to hybridisation with antibodies during blotting.

### Ats-1 protein toxicity and self-activating activity verification

The target gene bait vectors, pGBKT7-Ats-1, and pGADT7 AD empty vectors were co-transferred to the *MATα* strain by the LiAc method and coated on SD/–Leu/–Trp plates (Coolaber, Beijing, China). The monoclonal antibody (generally > 2 mm) with good growth on a SD/–Leu/–Trp (Coolaber, Beijing, China) plate was collected and placed into SD/–Leu/–Trp in liquid medium in shakers at 29 °C and 200 rpm for 200 h. The shaken bacterial droplets were aspirated to SD/–Ade/–His/–Leu/–Trp plates (Coolaber, Beijing, China). The growth of colonies on the plates was observed.

### Screening of the yeast two-hybrid library

The constructed vector plasmid pGBKT7-Ats-1 was used to transform the *MATα* strain by the LiAc method, and the strain was coated onto an SD/–Trp (Coolaber, Beijing, China) plate. The positive monoclonal yeasts were screened and inoculated into SD/-Trp medium (Coolaber, Beijing, China) and incubated overnight until the OD_600_ was > 1.5. An appropriate amount of the overnight bacterial solution was inoculated in YPDA (Coolaber, Beijing, China) and incubated until OD_600_ = 0.4–0.6. The bacterial liquid was collected and salmon sperm DNA (Coolaber, Beijing, China) was denatured twice. Then, LiAc (pH = 7.5) was added for re-suspension, and our laboratory yeast strain was added for the construction of the DNA library (pGADT7-cDNA) [30]. The denatured salmon sperm DNA (Coolaber, Beijing, China) was added and gently mixed. Then, LiAc was added, and the mixture was swirled vigorously. The yeast cells were collected by centrifugation, and YPD Plus liquid medium was added (Coolaber, Beijing, China). SD/-Leu/-Trp (Coolaber, Beijing, China) two-part plates and SD/–His/–Leu/–Trp/-Ade (Coolaber, Beijing, China) four-part plates were coated with 100 µl of the culture. Plaque growth was observed after 5–14 days of 30 °C culture.

Colonies greater than 2 mm on the four-part plates were chosen and spotted to SD/–His/–Leu/–Trp/–Ade (Coolaber, Beijing, China) plates. The colonies that had grown after 2–3 days were chosen for insert amplification PCR. The Primers used were 5’LD Amplimer: CTATTCGATGATGAAGATACCCCACCAAACCC and 3’LD Amplimer: GTGAACTTGCGGGGTTTTTCAGTATCTACGATT. PCR products were detected and sequenced.

### Co-transformation verification

Positive clones with successful sequencing were added to SD/-Leu liquid medium (Coolaber, Beijing, China) and incubated overnight. The plasmid was extracted using a yeast plasmid small amount extraction kit (Zomanbio, Beijing, China), and the plasmid was transformed into *DH5α*-competent cells and spotted on an LB plate. The clones were obtained and incubated overnight, and the plasmids were extracted using a plasmid small volume extraction kit (Zomanbio, Beijing, China). The plasmid obtained in this step and the pGBKT7-Ats-1 plasmid were each co-transformed into *Y2H gold* yeast competent cells, coated with SD/-Trp/-Leu dichotomized plate (Coolaber, Beijing, China), and cultured for three to five days, after which the colonies were observed and photographed. The monoclonal antibodies raised from the two deficient plates were chosen and added into SD/-Trp/-Leu deficient liquid medium (Coolaber, Beijing, China). After culturing, SD/-Trp/-Leu/-Ade/-His (Coolaber, Beijing, China) and SD/-Trp/-Leu/-Ade/-His (X-α-Gal) (Coolaber, Beijing, China) four deficient plates were coated. After 5–14 days, the colonies were observed and photographed.

### Co-immunoprecipitation (Co-IP)

The c-terminal of the interaction protein gene screened in the previous step was fused with a Myc tag and constructed into a pcDNA3.1 vector (Invitrogen, Carlsbad, CA, USA). The vectors were co-transfected with pcDNA3.1-Ats-1 (His) into HEK293T cells. After 48 h, native lysis buffer (Solarbio, Beijing, China) and protease inhibitor (Solarbio, Beijing, China) were added to extract the total protein. Mouse Anti-6× His tag antibody (Abcam, Cambridge, MA, USA) or Mouse Anti-Myc tag antibody (Abcam, Cambridge, MA, USA) was then added, and the solution was placed on a 120-rpm shaker at 8 °C for 3 h. Next, protein G-agarose (Roche, Mannheim, Germany) was added, and the solution was kept overnight at 8 °C and 120 rpm. The precipitate was collected by centrifugation the next day and washed three times with PBS (Solarbio, Beijing, China). Then, SDS-PAGE loading buffer (CWBIO, Beijing, China) was added, and the solution was boiled for 10 min prior to western blotting. And the blots were cut prior to hybridisation with antibodies during blotting. The primary antibody was mouse Anti-Myc tag antibody (Abcam, Cambridge, MA, USA) or mouse Anti-6× His tag antibody (1:1000) (Abcam, Cambridge, MA, USA), and Goat Anti-Mouse IgG H&L (Abcam, Cambridge, MA, USA) (1:3000) was used as the secondary antibody. A Super Signal™ West Femto Trial Kit (ThermoFisher, Waltham, MA, USA) was used to develop the solution.

### Bioinformatic analysis

The Immunomedicine Group online software (http://imed.med.ucm.es/Tools/antigenic.pl) and the RCSB PDB database (https://www.rcsb.org/) were used to predict the antigenic determinants and domains of Ats-1 and SDCBP. STRING software (https://version-11-0b.string-db.org/), the Gene Ontology database (GO, http://www.geneontology.org/), the Uniprot database (https://www.uniprot.org/), and the Kyoto Encyclopedia of Genes and Genome database (KEGG, http://www.genome.jp/kegg/) were used to analyze the potential functions and signaling pathways of SDCBP. Homology models for SDCBP, Ats-1, SDC1, SDC2, and SDC4 were generated using SWISS-MODEL (https://swissmodel.expasy.org/). Protein-protein docking was established with ClusPro 2.0 (https://cluspro.org/login.php).

### Quantitative real-time PCR (qRT-PCR) tests

The transfected cells were extracted with an Ultrapure RNA Kit (CWBIO, Beijing, China) for total RNA using a nucleic acid detector (Nanodrop ND-2000, USA) to determine the purity and concentration of RNA. A volume of 1 µL of RNA was reverse transcribed using an HIFiscript cDNA Synthesis Kit (CWBIO, Beijing, China) to synthesize cDNA, which was then quantified using the SYBR green dye method. Table S4 lists the primer sequences for SDCBP, EFNB1, IL5RA, IL5, NFASC, and 11 other genes. Glyceraldehyde 3-phosphate dehydrogenase (GAPDH) was used as the internal reference, and the primer sequences are shown in Table S4. The reaction system (10 µL) contained 5 µL UltraSYBR mixture, 0.2 µL upstream and downstream primers, 3.6 µL ddH_2_O, and 1 µL cDNA. The qRT-PCR reaction program was as follows: 95 °C denaturation for 5 s, extension at 95 °C for 5 s, and annealing at 60 °C for 30 s for 45 cycles, with a melting curve at 70–95 °C, 0.5 °C for 5 s. The PCR was performed using a quantitative PCR instrument (QuantStudio 5, USA).

### Immunoassay

The total protein of the cells transfected with the recombinant plasmid, pcDNA3.1-Ats-1, or with siRNA SDCBP was extracted using a native lysis buffer (Solarbio, Beijing, China) and protease inhibitor (Solarbio, Beijing, China). After the addition of SDS-PAGE Loading Buffer (CWBIO, Beijing, China), samples were boiled for 10 min at 100 °C, after which SDS-PAGE was performed using an 80 V constant pressure transfer membrane. The membranes were incubated with 5% skim milk powder for 3–4 h followed by primary and secondary antibodies for 2 h each. And the blots were cut prior to hybridisation with antibodies during blotting. A Super Signal™ West Femto Trial Kit (ThermoFisher, Waltham, MA, USA) was utilized for color development. Table S5 presents the details of the primary and secondary antibodies.

### Extraction and purification of exosomes

The empty pcDNA 3.1 vector, the pcDNA3.1-Ats-1 recombinant vector, and siRNA SDCBP + pcDNA 3.1-Ats-1 were transfected into HEK293T cells for 6 h, washed three times with PBS (Solarbio, Beijing, China), and incubated in the fresh cell medium complemented with 10% exosome-depleted serum System Biosciences (Palo Alto, CA, USA) for 48 h. After this, 60 mL of cell supernatant was collected and placed on ice.

After centrifugation at 3000 g for 10 min at 4 °C, the cell debris was removed from the supernatant and transferred to a new centrifuge tube. Then, 15 mL of exosome concentration solution (Umibio, Shanghai, China) was added, vortexed for 1 min, and then kept at a temperature between 2 and 8 °C for 2 h. Then, the sample was centrifuged at 10,000 g at 4 °C for 60 min, after which the supernatant was discarded and the precipitate was saved. One hundred microliters of PBS (Solarbio, Beijing, China) was used to dissolve the precipitate, and the solution was transferred to a 1.5 mL centrifuge tube. The tube was centrifuged at 12,000 g for 2 min at 4 °C and the supernatant was retained because it was rich in exosome particles. The supernatant obtained after centrifugation was added into the upper chamber of an Exosome Purification Filter (Umibio, Shanghai, China) and centrifuged at 3000 g, 4 °C for 10 min, leaving the liquid portion at the bottom of the EPF column; this contained the purified exosome particles. The purified exosomes were stored in a refrigerator at − 80 °C for subsequent experiments.

### Transmission electron microscopy

The freshly purified exosome sample was mixed with 100 µL of 2% PFA (Sigma Aldrich, St. Louis, MO, USA). Then, 5 µL of the mixed solution was added to a Formvar-carbon sample-loaded copper mesh (PELCO, Fresno, CA, United States), and 100 µL of PBS was added to the sealing membrane. Subsequently, the copper mesh (with the Formvar membrane side down) was successively covered with PBS droplets (for washing) and 50 µL of 1% glutaraldehyde solution (Ted Pella, Redding, CA, United States) for 5 min, 100 µL of ddH_2_O for 2 min (eight washes), 50 µL of uranium dioxide droplets for 75 min, and 50 µL of methylcellulose (Sigma Aldrich, St. Louis, MO, USA) for 10 min (on ice). The sample was air-dried for 5–10 min and photographed under an electron microscope at 80 kV using a transmission electron microscope (Jeol, Tokyo, Japan).

### Nanoparticle tracking analysis

Appropriate exosome samples were diluted with PBS buffer (Solarbio, Beijing, China). The particle size and concentration of exosomes were measured through nanoparticle tracking analysis (NTA) at Vivacell Biosciences using the ZetaView PMX 110 (Particle Metrix, Meerbusch, Germany) and the corresponding ZetaView 8.04.02 software. We measured and collected NTA results at a total of 11 locations. The ZetaView system was calibrated using 110 nm polystyrene particles, and the temperature was maintained between approximately 23 and 30 °C.

### Expression of marker proteins of exosomes was detected by western blotting

A proper amount of purified exosome was mixed with lysis solution and placed on ice for 30 min. A working solution and dilution standard were prepared according to the instructions of a BCA protein quantification kit (Sigma Aldrich, St. Louis, MO, USA). Then, 25 µL of the standard and 25 µL of the lysed sample were added to the wells of a 96-well plate and 200 µL of BCA Working Solution was added to each well and gently mixed with shaking. The 96-well plates were incubated for 30 min in an incubator at 37 °C. The absorbance at 570 nm was measured by a multifunctional microplate reader (Perkin Elmer, VICTOR X), and the protein concentration was calculated according to the standard curve.

A proper amount of exosome samples was added into the loading buffer, mixed, and boiled at 100 °C for 10 min. Based on the protein concentration quantified using BCA protein, the loading volume of each sample for the same total protein amount was calculated. SDS-PAGE was performed, and different volumes of samples were added for electrophoresis at 120 mA for 90 min. The protein was electrotransferred to a PVDF membrane at 300 mA for 60 min in an ice bath. The PVDF membrane was blocked in blocking solution (containing 10% defatted milk TBST solution) and developed using TBST solution diluted primary antibody (1:1000) for 2 h each. And the blots were cut prior to hybridisation with antibodies during blotting. Solutions A and B were mixed in a 1:1 ratio for coloration using a Super Signal™ West Femto Trial Kit (ThermoFisher, Waltham, MA, USA). Table S5 presents the details of primary and secondary antibodies.

### Data analysis

All data presented herein represent the results from three separate experiments and are presented as the mean ± SD. GraphPad Prism software was used to draw the figures in this paper. SPSS software was used for statistical analysis, and immunoblotting results were semi-quantified using ImageJ software. Data were compared with different groups using one-way analysis of variance (ANOVA), Student–Newman–Keuls (SNK) tests, and Student’s *t*-tests.

## Results

### Expression of Ats-1 in HEK293T cells

**Fig. 1:**
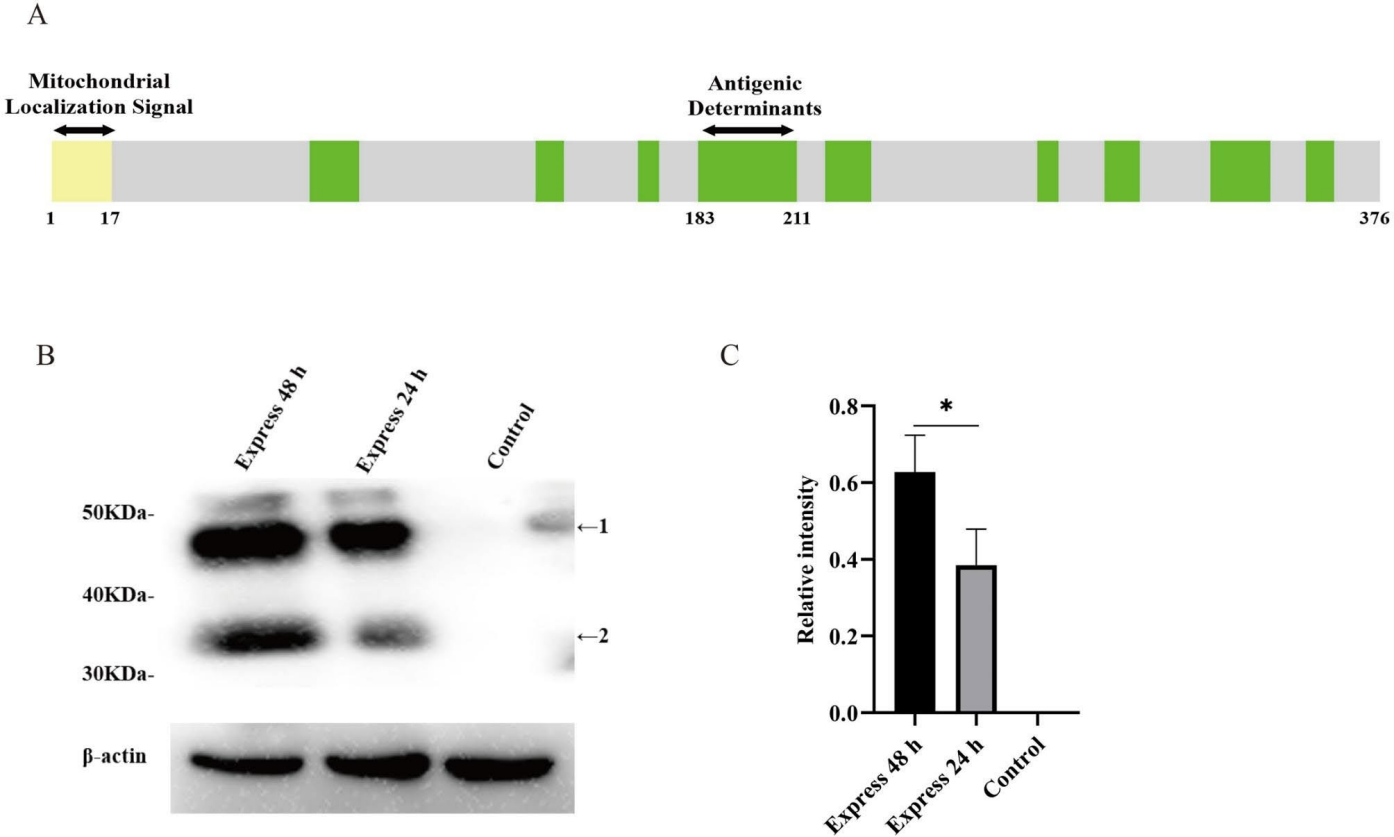
Ats-1 structure prediction and expression. **A**. Schematic diagram of the Ats-1 protein structure, where light yellow indicates the mitochondrial localization signal, and green indicates the antigenic determinant. Ats-1 had a total of 10 antigenic determinants. The numbers indicate the amino acid sites. The specific amino acid sites of each protein structure are provided in Supplementary Fig. 1. **B**. Expression of Ats-1 in HEK293T cells. After pcDNA3.1-Ats-1 was transferred to the HEK293T cells, samples were collected for Western blot analysis at 24 and 48 h. The blots were cut prior to hybridisation with antibodies during blotting. **C**. Western blot results were semi-quantitatively analyzed using ImageJ software. All data are shown as mean ± SEM from three independent tests. *p < 0.05

Ats-1 is a secreted protein of *A. phagocytophilum*, and its N-terminal end has a mitochondrial positioning signal that can target the mitochondria, where the targeting sequence is then cut. The mitochondrial localization signals consisted of N-terminal amino acids 1–17 (Fig. 1A). Therefore, Ats-1 in host cells has two forms of expression: natural Ats-1 in the cytoplasm (48 kDa) and cleaved Ats-1 in the mitochondria (35 kDa) [23]. In addition, the Ats-1 protein had 10 antigenic determinants (Fig. 1A), and the specific amino acid sites are shown in Fig. S1 A. Western blot results (Fig. 1B) showed that after transfection of the recombinant plasmid pcDNA 3.1-Ats-1 into cells, Ats-1 expressed two sizes of proteins, 48 kDa and 35 kDa. With the prolongation of transfection time, the expression of the Ats-1 protein gradually increased (Fig. 1B). In the semi-quantitative analysis (Fig. 1C), Ats-1 protein expression was the highest after 48 h of transfection, and the following experiments all used the total protein being transfected for 48 h. These results indicated that the transfection model was successfully established, and the Ats-1 protein was smoothly expressed in HEK293T cells.

### Potential interaction proteins screened by the yeast two-hybrid and co-transformation experiments

**Fig. 2:**
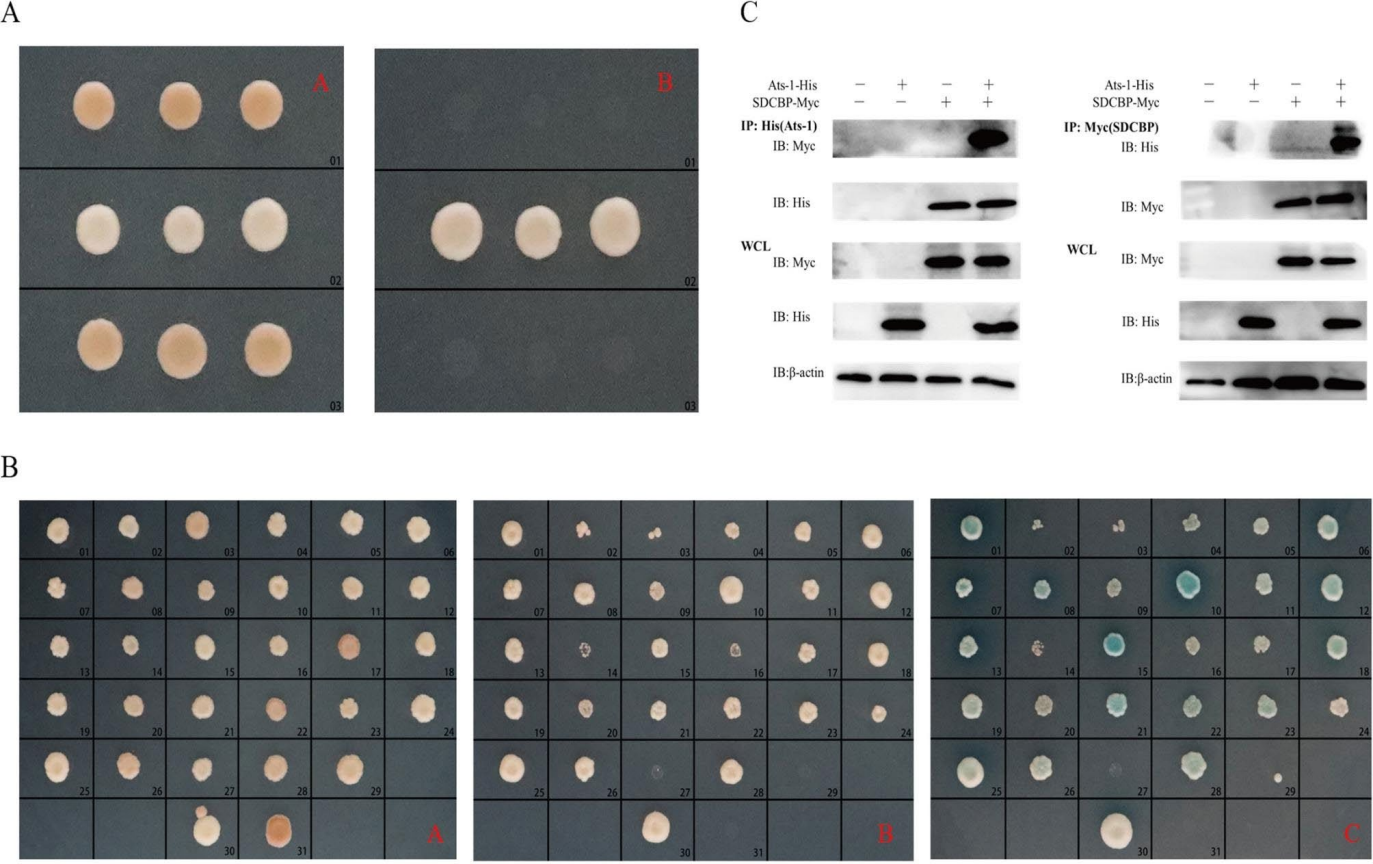
Verification and analysis of the Ats-1 interaction protein SDCBP. **A**. Ats-1 protein self-activating activity verification. 01, negative control. 02, positive control. 03, Ats-1-pGBKT7/pGADT7. (A-A) Growth on the SD/–Leu/–Trp (DDO) medium. (A-B) Growth on the SD/–Ade/–His/–Leu/–Trp (QDO) medium. The results in the figure were obtained from three independent replicate experiments. **B**. Transfer back verification test of potential interacting proteins with Ats-1 protein. (B-A) Growth on the DDO medium. (B-B) Growth on the QDO medium. (B-C) Growth on the SD/–Ade/–His/–Leu/–Trp/X-α-gal (QDO/X) medium. 01–31, additional details of potential interaction proteins are provided in Table S4. The results in the figure were obtained from three independent replicate experiments. **C**. Interaction between Ats-1 and SDCBP. The plasmids encoding Ats-1-His and SDCBP-Myc were co-transfected into the HEK293T cells. After 48 h, the cells were collected and the whole-cell lysates were immunoprecipitated with anti-His antibody (left) or anti-Myc antibody (right) and protein A/G agarose beads. Ats-1 and SDCBP were then detected with Western blotting assays using the antibodies against either the His or Myc tags. The blots were cut prior to hybridisation with antibodies during blotting. The relative intensities for the Western blots were semi-quantified using ImageJ software and are presented as bar graphs in Fig. S2, where the results in the figure were obtained from three independent replicate experiments

It has been reported that a fraction of intracellular Ats-1 is localized in the mitochondria, while most Ats-1 is secreted into the cytoplasm [23]. It is important to study the biological functions of cytoplasmic Ats-1 and the interaction with host cell proteins. Using the DNA library constructed in the laboratory, we screened for interacting proteins of Ats-1 by the yeast two-hybrid technique. In the first step, we needed to verify whether the target protein Ats-1 was toxic or self-activating. Ats-1 had white colonies on SD/–Leu/–Trp plates and sterile colonies on SD/–Ade/–His/–Leu/–Trp plates (Fig. 2A). This indicated that the target protein Ats-1 was non-toxic and non-self-activating and could be used for library screening. After the yeast *MATα* strain was co-transformed with pGBKT7-Ats-1 and pGADT7-cDNA plasmids, it was screened using a variety of defective media followed by positive PCR and sequencing. A total of 23 potential proteins (Table S1) were identified as interacting with Ats-1. To prevent a false-positive result, we constructed the sequences of these 23 potential interaction proteins on the pGADT7 vector and co-transformed the yeast *MATα* strain with the pGBKT7-Ats-1 plasmid for screening auxotrophic plates. A total of 19 potential interacting proteins were screened by co-transformation verification (Fig. 2B).

### Interaction protein SDCBP re-screened by co-immunoprecipitation

To further verify whether the 19 potential interacting proteins actually interacted with the target protein, we performed a co-immunoprecipitation experiment. From the experimental results (Fig. 2C), the presence of Ats-1 and SDCBP could be detected when the antibodies of His Tag and Myc Tag were used in immunoblotting (IB). The semi-quantitative analysis results of western blotting are shown in the form of a histogram in Fig. S2, illustrating that SDCBP interacted with Ats-1. Thus, the only truly interacting protein, SDCBP, was verified.

### Analysis of potential biological function of SDCBP

**Fig. 3:**
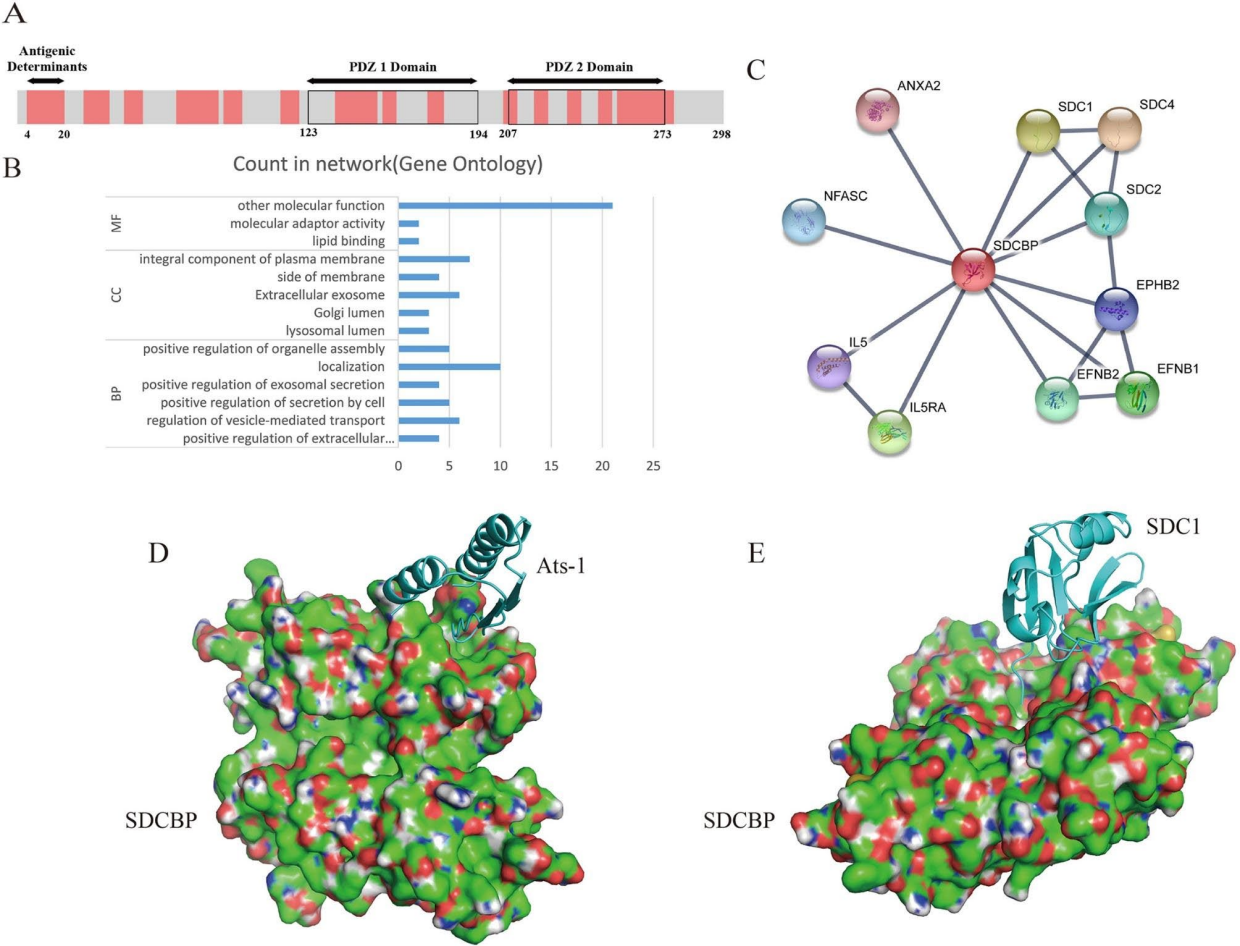
Bioinformatic analysis of SDCBP. **A**. Schematic diagram of the SDCBP structure. The antigenic determinants and domains of SDCBP were predicted using Immunomedicine Group online software and the RCSB PDB database. Red indicates antigenic determinants. SDCBP had 16 antigenic determinants in total. Black indicates the PDZ domain, and the numbers indicate amino acid sites. Detailed amino acid sites for each construct are shown in Supplementary Fig. 2. **B**. SDCBP GO term analysis. BP: Biological Process. CC: Cellular Component. **C**. SDCBP PPI analysis. The line thickness indicates the strength of data support. **D**. Homology models for SDCBP and Ats-1 were built by SWISS-MODEL. Protein-protein docking between SDCBP and Ats-1 was established with ClusPro 2.0. **E**. Homology models for SDCBP and SDC1 were built by SWISS-MODEL. Protein-protein docking between SDCBP and SDC1 was established with ClusPro 2.0

The potential functions of SDCBP were analyzed using multiple online software and database resources to guide the next step of SDCBP functional validation. SDCBP protein structures containing 10 antigenic determinants and 2 PDZ domains (Fig. 3A) were analyzed using the Immunomedicine Group online software and the RCSB PDB database. SDCBP was bound to other proteins via the PDZ domain. Structurally specific amino acid sites are shown in Fig. S1 A and B.

Gene ontology (GO) analysis showed that SDCBP was involved in the positive regulation of the extracellular exosome assembly and extracellular exosome (Fig. 3B). We also noticed that SDCBP was involved in the syndecan binding (Table S2) process in other molecular functions (MFs). In addition, SDCBP was involved in the exosome pathway (Table 1), and these processes were associated with exosome secretion processes. Meanwhile, in the protein-protein interaction (PPI) network, there were some interconnected proteins that could cooperate with SDCBP to regulate exosome biogenesis (Fig. 3C), such as SDC1, SDC2, and SDC4.

**Table 1:** Syntenin-1 (SDCBP) KEGG Pathway

Pathway	Description
hsa05144	Malaria
hsa04514	Cell adhesion molecules
hsa05418	Fluid shear stress and atherosclerosis
hsa05205	Proteoglycans in cancer
hsa04147	Exosome

Ats-1, SDCBP, SDC1, SDC2, and SDC4 were subjected to homology modeling and protein docking using SWISS-MODEL and ClusPro 2.0. SDCBP could interact with Ats-1 (Fig. 3D), which was consistent with previous experimental results. SDCBP could also bind to SDC1 (Fig. 3E), SDC2 (Fig. S1C), and SDC4 (Fig. S1 D), which was consistent with the GO (MF) results for SDCBP. From these data, we concluded that SDCBP was involved in the secretion process of exosomes and that it affected the secretion of exosomes by binding to interacting proteins (SDC1\2\4).

### Ats-1 upregulates the expression of SDCBP and its related proteins in HEK293T cells

**Fig. 4:**
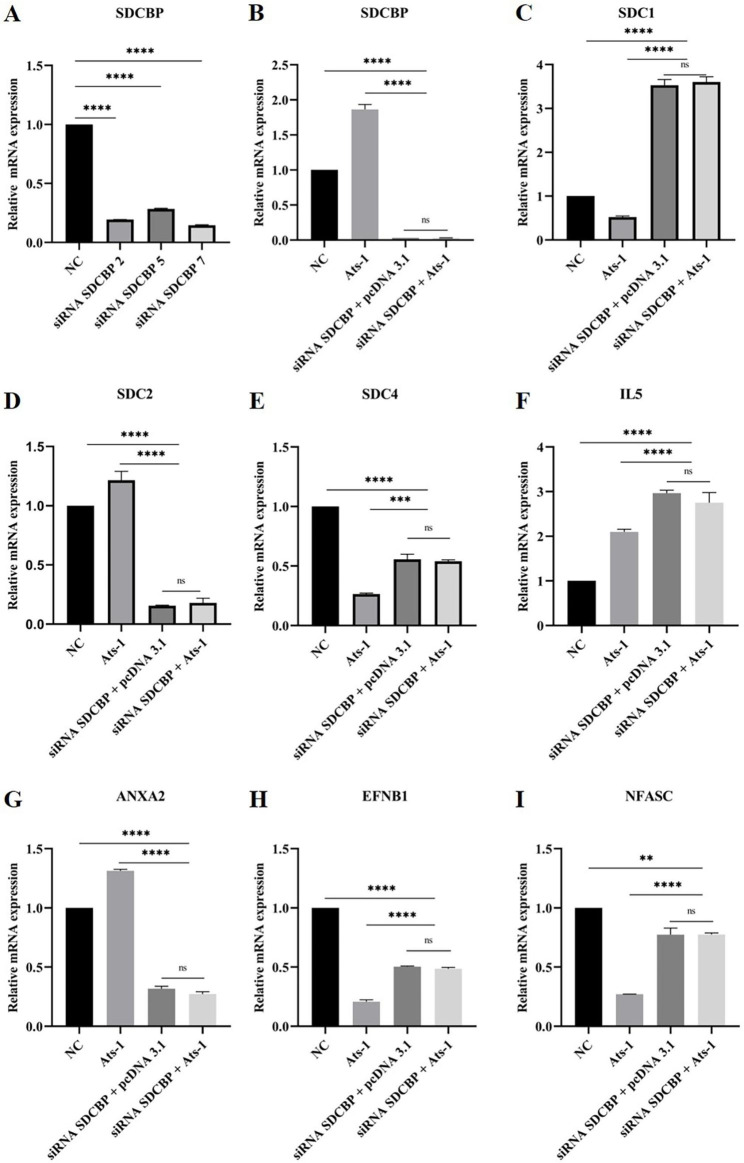
qPCR of the expression levels of SDCBP and its related proteins induced by Ats-1. siRNA SDCBP was transferred into the HEK293T cells, and 24 h later, pcDNA 3.1 or Ats-1 was transferred into the HEK293T cells. After 48 h, total cellular RNA was extracted and reverse transcribed into cDNA for qPCR analysis. **A**. mRNA expression level of SDCBP after siRNA SDCBP 2/5/7 transfer. **B**. mRNA expression level of SDCBP before and after siRNA SDCBP /Ats-1 transfer. **C**. mRNA expression level of SDC1 before and after siRNA SDCBP/Ats-1 transfer. **D**. mRNA expression level of SDC2 before and after siRNA SDCBP/Ats-1 transfer. **E**. mRNA expression level of SDC4 before and after siRNA SDCBP/Ats-1 transfer. **F**. mRNA expression level of IL5 before and after siRNA SDCBP/Ats-1 transfer. **G**. mRNA expression level of ANXA2 before and after siRNA SDCBP/Ats-1 transfer. **H**. mRNA expression level of EFNB1 before and after siRNA SDCBP/Ats-1 transfer. **I**. mRNA expression level of NFASC before and after siRNA SDCBP/Ats-1 transfer. All data are shown as mean ± SD from three independent tests. ns, no significant difference, *p < 0.05, **p < 0.01, ***p < 0.001, ****p < 0.0001

**Fig. 5:**
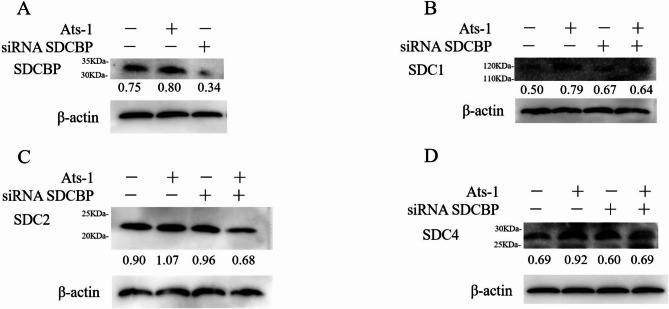
Western blotting of the expression levels of SDCBP and some related proteins induced by Ats-1. siRNA SDCBP was/was not transfected into the HEK293T cells, and Ats-1 was transfected into HEK293T cells 24 h later. After 48 h, the cells were lysed to obtain the total protein. Western blot was performed using the corresponding primary and secondary antibodies (Table S3). The blots were cut prior to hybridisation with antibodies during blotting. **A**. Expression levels of SDCBP before and after siRNA SDCBP/Ats-1 expression. **B**. Expression levels of SDC1 before and after siRNA SDCBP/Ats-1 expression. **C**. Expression levels of SDC2 before and after siRNA SDCBP/Ats-1 expression. **D**. Expression levels of SDC4 before and after siRNA SDCBP/Ats-1 expression. ImageJ analysis of the level of Western blotting is shown in terms of the normalized values, where the presented values represent the results of three independent experiments

How does Ats-1 affect the expression of SDCBP in host cells? Three SDCBP-targeting siRNAs (siRNA SDCBPs) were designed and synthesized (Table S3). Of these, siRNA SDCBP 7 could reduce the RNA expression of SDCBP by 89%, obtaining the best interference effect (Fig. 4A). In the latter assay, the siRNA SDCBP was siRNA SDCBP 7. After the expression of Ats-1, the RNA level of SDCBP in HEK293T cells increased significantly (Fig. 4B). We detected the protein expression of SDCBP by western blotting. SDCBP was detected in pcDNA3.1, Ats-1, and siRNA SDCBP + Ats-1 (Fig. 5A). Semi-quantitative analysis of the western blot results by ImageJ revealed that the protein expression level of SDCBP (Fig. 5A) was consistent with the trend of RNA expression (Fig. 4B), indicating that Ats-1 significantly upregulated the expression level of SDCBP.

From the analysis of the PPIs, 10 proteins associated with SDCBP were found. The RNA levels of these 10 associated proteins following Ats-1 expression were examined by quantitative real-time PCR (qRT-PCR). Following Ats-1 expression, the mRNA levels of SDC2 (Fig. 4D), ANXA2 (Fig. 4G), IL5 (Fig. 4F), and IL5RA (Fig. S3 C) were upregulated, while those of SDC1 (Fig. 4C), SDC4 (Fig. 4E), EFNB1 (Fig. 4H), NFASC (Fig. 4I), EFNB2 (Fig. S3 A), and EPHB2 (Fig. S3 B) were downregulated. In siRNA SDCBP + pcDNA 3.1 and siRNA SDCBP + Ats-1, compared to Ats-1, the RNA levels of these 10 associated proteins showed significant differences, but there was no difference between individual RNA levels.

In combination with GO and KEGG analyses, we selected the secretion of exosomes as the pathway of interest for subsequent functional verification. Three of the 10 associated proteins (SDC1, SDC2, and SDC4) were associated with exosome secretion. Next, we detected the protein expression of SDC1 (Fig. 5B), SDC2 (Fig. 5C) and SDC4 (Fig. 5D) through western blotting and performed semi-quantitative analysis on the western blot results using ImageJ. The results showed that the expression levels of these three proteins in Ats-1 were upregulated compared with those in pcDNA3.1. Both siRNA SDCBP + pcDNA 3.1 and siRNA SDCBP + Ats-1 were downregulated compared with Ats-1. The protein expression levels of SDC1 and SDC4 in cells were not consistent with the trends in mRNA levels. However, studies have shown that post-transcriptional control, translational control, and degradation control play a significant role in protein concentration determination [31]. Therefore, there may not be a corresponding relationship between the protein level and the mRNA level. In our future research, we will verify the protein function, as the protein level results are the most important. Altogether, these results suggest that following Ats-1 expression, the protein expression of SDC1, SDC2, and SDC4 was upregulated through interactions with SDCBP.

### Ats-1 promotes exosome secretion in HEK293T cells

**Fig. 6:**
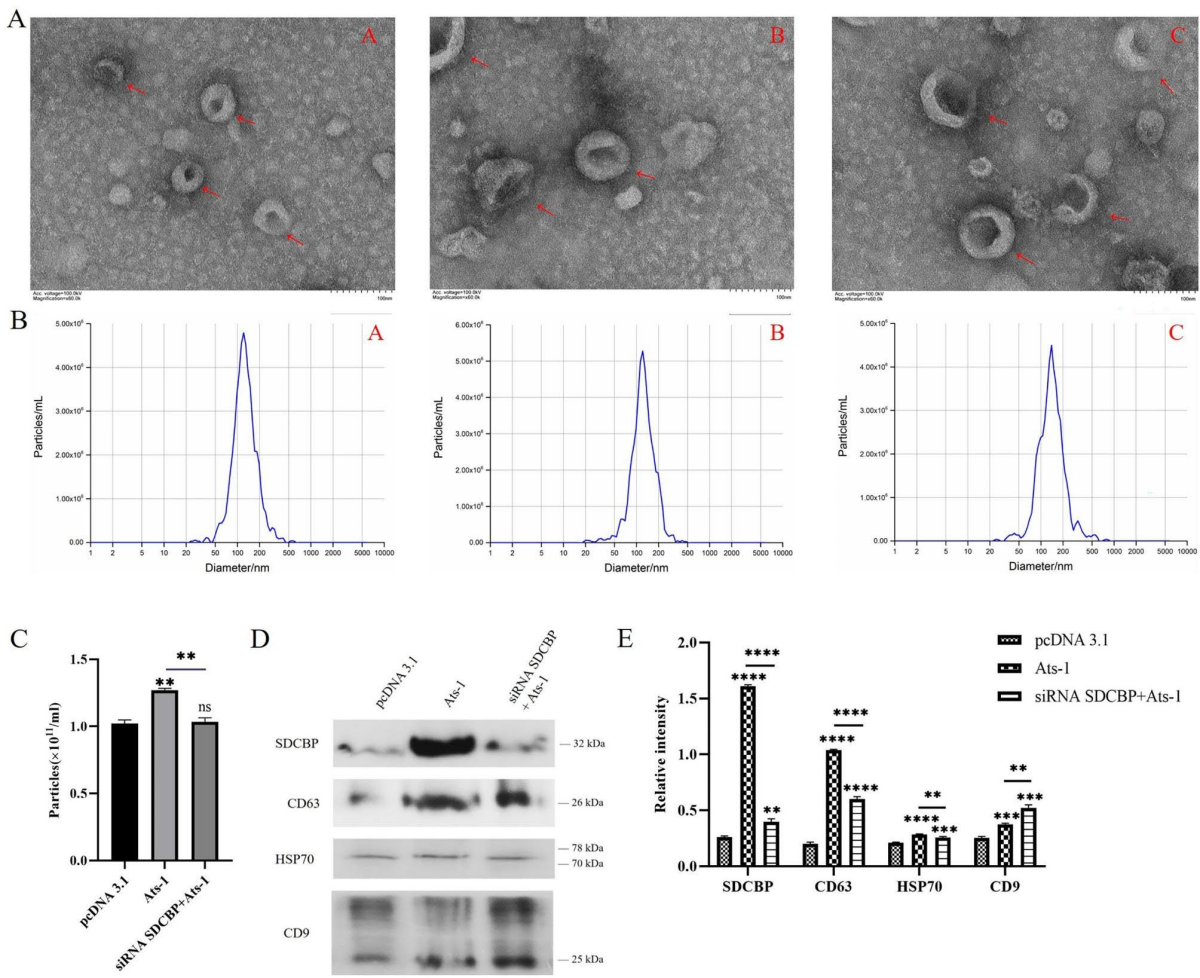
Isolation and identification of exosomes. **A**. Representative electron microscopy images are shown. Scale bar = 100 nm. ←, exosomes. (A-A) Electron microscopy results of the pcDNA 3.1 cell pellet. (A-B): Electron microscopy results of the Ats-1 cell pellet. (A-C) Electron microscopy results of the siRNA SDCBP + Ats-1 cell pellet. **B**. Representative nanoparticle tracking analysis (NTA) traces of exosomes derived, normalized to the cell number. (B-A) NTA traces of exosomes derived from the pcDNA 3.1 cells. (B-B) NTA traces of exosomes derived from the Ats-1 cells. (B-C) NTA traces of exosomes derived from the siRNA SDCBP + Ats-1 cells. **C**. NTA quantification of three independent experiments. **D**. Western blot analysis of microvesicles purified from equal numbers of the pcDNA 3.1, Ats-1, and siRNA SDCBP + Ats-1 cells. The blots were cut prior to hybridisation with antibodies during blotting. The results in the figure were obtained from three independent replicate experiments. **E**. Quantification of the exosomal protein levels in the microvesicles obtained from the pcDNA 3.1, Ats-1, and siRNA SDCBP + Ats-1 cells in the three independent experiments. All data are shown as mean ± SD from three independent tests. ns, no significant difference, **p < 0.01, ***p < 0.001, ****p < 0.0001

According to the bioinformatic analysis, SDCBP, SDC1, SDC2, and SDC4 were all related to exosome secretion. Therefore, if the expression levels of SDCBP, SDC1, SDC2, and SDC4 increase, the content of exosomes may increase. To verify this hypothesis, we extracted and identified exosomes from the cell supernatant. In the transmission electron microscope results, the microvesicles extracted from the supernatant of HEK293T cells transfected with pcDNA 3.1, Ats-1, and siRNA SDCBP + Ats-1 had two-sided membrane structures (Fig. 6A). The NTA results showed that the size distributions of microvesicles in the supernatants of pcDNA 3.1, Ats-1, and siRNA SDCBP + Ats-1 transfected HEK293T cells were about 119.2, 118, and 133.6 nm in diameter (Fig. 6B), respectively. The majority had a size similar to those generally described for exosomes (30–150 nm) [32]. Microvesicles detected from NTA and electron microscopic analyses revealed that the cell supernatants were exosomes. The NTA results also showed that the Ats-1 cells secreted fewer exosomes compared to the pcDNA 3.1 cells (Fig. 6C), indicating that Ats-1 expression induced exosome secretion in the HEK293T cells. Compared to the Ats-1 cells, the siRNA SDCBP + Ats-1 cells secreted reduced numbers of exosomes, indicating that silencing SDCBP resulted in decreased exosome secretion by the HEK293T cells.

To further examine the number of exosomes in the cell supernatants, widely recognized exosome marker proteins in supernatant pellets from the three groups of transfected cells were analyzed via western blotting. Exosome marker proteins SDCBP, CD63, HSP70, and CD9 were found to be very abundant in HEK293T cell supernatants [33]. According to the calculation by BCA protein, the exosome protein concentrations in the supernatant of HEK293T cells transfected with pcDNA 3.1, Ats-1, and siRNA SDCBP + Ats-1 were greater than 200 ng/µL, indicating that they could be used for subsequent experiments. The western blot results (Fig. 6D) were quantitatively analyzed using ImageJ (Fig. 6E). Compared to the pcDNA 3.1 pellet, SDCBP, CD63, HSP70, and CD9 expression was significantly increased in the Ats-1 pellet, indicating that Ats-1 expression induced exosome secretion in the HEK293T cells. SDCBP, CD63, and HSP70 expression was significantly lower in the siRNA SDCBP + Ats-1 pellet compared to that in the Ats-1 pellet, indicating that silencing SDCBP resulted in decreased exosome secretion by the HEK293T cells. This result was consistent with the NTA results, confirming that Ats-1 induced exosome secretion in the HEK293T cells via SDCBP. However, after silencing SDCBP, Ats-1 overexpression increased CD9 in the cell supernatant, possibly because Ats-1 upregulated CD9 secretion by regulating other pathways.

### Ats-1 mediated secretion via the exosome pathway of the host cells

**Fig. 7:**
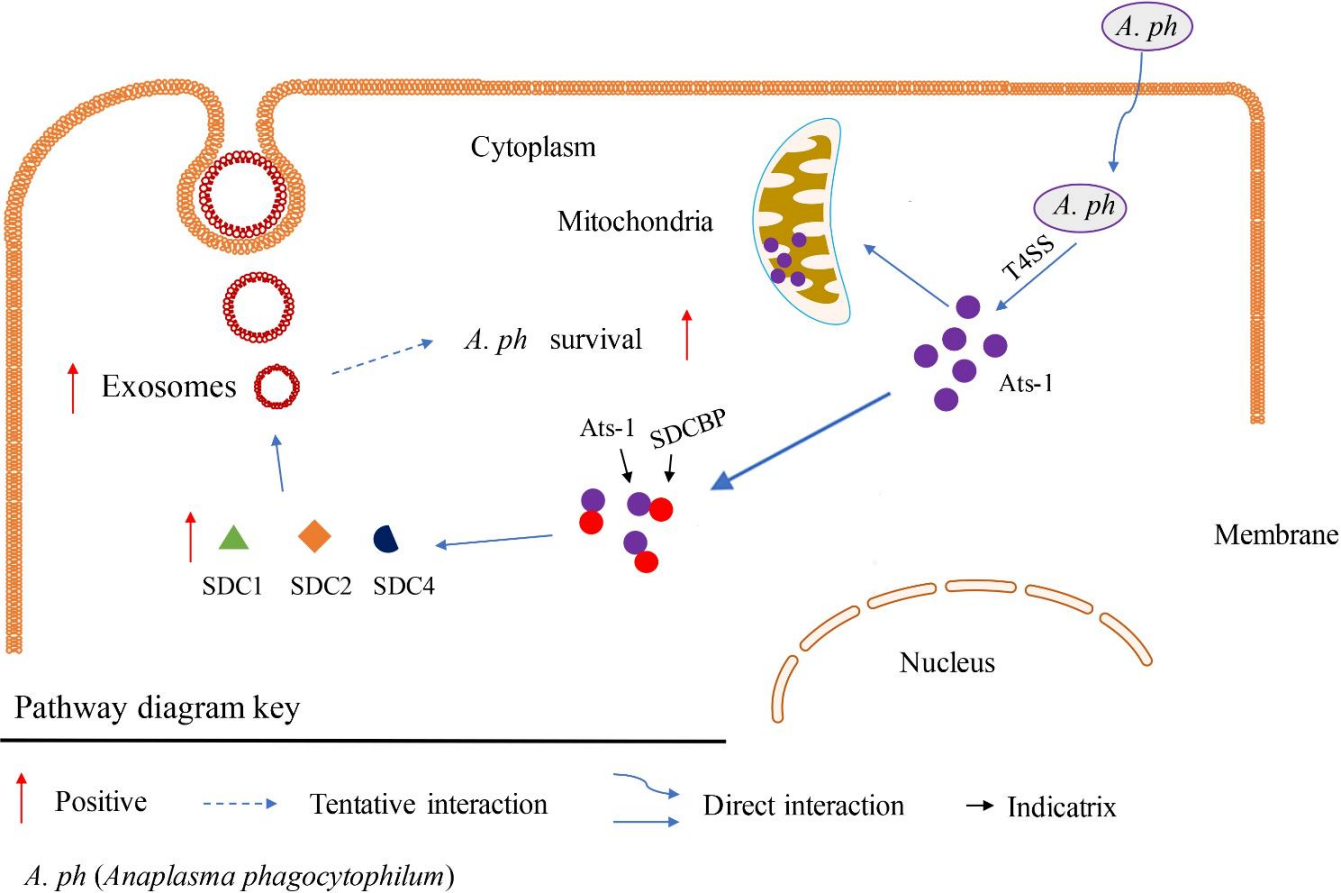
Overview of the path for Ats-1 participation. The solid line represents the known results and the dashed line represents the potential results

Based on the above results, we can summarize the pathways involving Ats-1. *Anaplasma phagocytophilum* infects host cells and secretes the effector protein Ats-1 (48 kDa) through T4SS, which interacts with SDCBP in HEK293T cells. The expression levels of proteins regulating exosome biogenesis, SDC1, SDC2, and SDC4 increase, along with the secretion of exosomes. This in turn affects the transmission of information between the host cell and the receptor cell, which may be conducive to the survival of *A. phagocytophilum* in the host cell. To concisely summarize the results of the study, a pathway profile was drawn (Fig. 7).

## Discussion

Ats-1 was first discovered by Niu et al. [23]. The protein is able to target mitochondria in host cells to inhibit etoposide-induced programmed cell death and thus increase the survival time of *A. phagocytophilum* in host cells. This study revealed that *A. phagocytophilum* effector protein Ats-1 upregulated the expressions of proteins regulating exosome biogenesis, SDC1, SDC2, and SDC4 through interaction with SDCBP, thereby regulating the secretion of exosomes in HEK293T cells. This is a new discovery in the study of the action mechanism of Ats-1 in host cells and provides a new direction for the study of the pathogenic molecular mechanism of *A. phagocytophilum*.

SDCBP is an intracellular scaffold protein that contains the PDZ domain, and as a result can bind to a variety of proteins and participate in the regulation of biological processes such as protein transport, cancer cell metastasis, exosome secretion, and synapse formation. The interaction of CD63 with SDCBP is very important for the transport of *human papillomavirus* after its endocytosis into host cells, and this can control the transmission of virus particles to multivesicular endosomes [34]. SDCBP in combination with UNC93B1 regulates the activation and signal transduction of Toll-like receptor 7 [35]. In addition, the interaction between SDCBP and NG2 participates in the migration of oligodendroglial precursors and plays an important role [36]. The above studies have shown that SDCBP can exert different biological functions in host cells by virtue of its coupling with different proteins. Our results indicate that the binding of SDCBP to Ats-1 promotes the secretion of exosomes from HEK293T cells, which may be beneficial for persistent intracellular infection of *A. phagocytophilum.*

The exosome is a single-membrane vesicle derived from endosomal origin with a diameter of about 30–150 nm [32]. Multivesicular bodies (MVBs), which are late endosomes, bud intracellularly to produce intraluminal vesicles (ILVs), which are exosomes [37]. Syntenin (SDCBP) has a PDZ domain and three N-terminal LYPXnL motifs through which it can interact directly with the domains of syndecans [38, 39]. SDC1, SDC2, and SDC4 are members of the syndecan family and can interact with SDCBP [40]. In our results, Ats-1 upregulated the expression of SDCBP in HEK293T cells and simultaneously increased the expression levels of SDC1, SDC2, and SDC4. After SDCBP knockdown, the expression levels of SDC1, SDC2, and SDC4 decreased significantly, indicating that Ats-1 increased the expression levels of SDC1, SDC2, and SDC4 through interaction with SDCBP. This may have been achieved by an increase in the formation of the syndecan–syntenin–Alix complex and the induction of MVBs to bud and produce ILVs. We determined through NTA that Ats-1 could increase the concentration of exosomes in the cell supernatant, and the expression concentration of exosomes decreased after SDCBP was knocked down. This demonstrated that Ats-1 could increase the production of exosomes through interaction with SDCBP.

Exosomes contain large amounts of microRNA, mRNA, lipids, and protein that are released outside of the cells for intercellular communication [41, 42]. When exosomes come into contact with receptor cells, exosome protein-mediated cell signaling stimulates receptor cells, and proteins, microRNAs, or mRNAs transmitted by exosomes change the dynamics of receptor cells [43]. The primary tumor cells and distant organs communicate with each other through exosomes and microvesicles, and this communication is crucial for the metastasis of tumor cells [44]. It has been found that programmed death-ligand 1 (PD-L1) carried by exosomes can interact with the programmed death-1 (PD-1) receptor on the T cell surface to achieve immunosuppression [45]. In this study, Ats-1 expression induced an increase in the number of exosomes secreted by HEK293T cells. Further experimental research is needed to examine the role these exosomes play in the cell microenvironment and whether they can cause immunosuppression. This effect that would be beneficial to the survival and proliferation of *A. phagocytophilum*.

A recent study has provided us with a comprehensive quantitative atlas of prevalent exosome proteins [43]. The data show that the abundances of CD9, CD63, and CD81 proteins in the exosome differ according to the cell type, while SDCBP, Alix, and TSG101 are prevalent in all exosomes. SDCBP is the most abundant protein in the exosome, and it can be used as a universal marker for the identification of exosomes. In previous studies, CD9, CD63, and CD81 were mostly used as identification markers, while the work of Kugeratski [33] provided a new direction for exosome identification technology. In this study, we identified purified exosomes with high levels of SDCBP and CD63 and low levels of CD9 and Hsp70 when four exosomes were selected. The results were consistent with those of Kugeratski, indicating that SDCBP and CD63 could be used as biomarkers for the exosomes secreted by HEK293T cells.

It has been reported that after secretion in host cells, Ats-1 targets the mitochondria to inhibit apoptosis [23]. Our recent study found that the expression of Ats-1 upregulated five proteins (NDUFB3, NDUFB5, NDUFS7, COX6C, and SLC25A5) involved in the mitochondrial respiratory chain, leading to increased ATP synthesis, enhanced cellular proliferation, and decreased apoptosis rates [24]. This study revealed that Ats-1 interacted with SDCBP in HEK293T cells, regulated the secretion of exosomes, and affected the signal transduction of infected host cells and other cells. These effects may ultimately be beneficial to the survival and proliferation of *A. phagocytophilum* in host cells.

## Conclusion

All of the above data suggest that we have found a mechanism for the action of *A. phagocytophilum* effector protein Ats-1 in the cytoplasm. In host cells, we identified SDCBP, a novel interacting protein of Ats-1. Through interactions between Ats-1 and SDCBP, the expression levels of SDCBP, SDC1, SDC2, and SDC4, which regulate the biological process of exosomes, were upregulated and the secretion of exosomes in HEK293T cells was induced. This could enhance cell signaling between infected cells and recipient cells. These findings have added new content to the functional study of Ats-1 and have provided a new direction for studying the pathogenesis of *A. phagocytophilum*.

### Electronic supplementary material

Below is the link to the electronic supplementary material.

Supplementary Material 1

## Data Availability

The datasets used and analyzed during the current study are available from the corresponding author on reasonable request.
