# Supplementary material for: Anaplasma phagocytophilum Ats-1 enhances exosome secretion through Syntenin-1
Source: BMC Microbiol. 2023 Sep 27;23:271. doi: 10.1186/s12866-023-03023-4 (PMC10523776; doi:10.1186/s12866-023-03023-4)
Supplement: Supplementary file 1 — Supplementary Material 1 [file 12866_2023_3023_MOESM1_ESM.pdf]

Supplementary materials

Table S1. Co-transformation verified the corresponding genes in Fig 2

Number	Gene	Gene ID
1	Homo sapiens SERTA domain containing 1 (SERTAD1), mRNA	NM_013376.4
2	Homo sapiens cDNA FLJ75708 complete cds, highly similar to Homo sapiens N-myc (and STAT) interactor (NMI), mRNA	AK291548.1
3	Homo sapiens histocompatibility minor 13 (HM13), transcript variant 3, mRNA	NM_178581.3
4	Homo sapiens keratin 18 (KRT18), transcript variant 1, mRNA	NM_000224.3
5	Homo sapiens MAGE family member A6 (MAGEA6), transcript variant 2, mRNA	NM_175868.4
6	Homo sapiens filamin A (FLNA), transcript variant 2, mRNA	NM_001110556.2
7	Homo sapiens four and a half LIM domains 2 (FHL2), transcript variant 5, mRNA	NM_001039492.3
8	Homo sapiens serine and arginine rich splicing factor 2 (SRSF2), RefSeqGene (LRG_640) on chromosome 17	NG_032905.1
9	Homo sapiens keratin 18 (KRT18), transcript variant 1, mRNA	NM_000224.3
10	Homo sapiens fascin actin-bundling protein 1 (FSCN1), mRNA.	NM_003088.4
11	Homo sapiens vimentin, mRNA (cDNA clone MGC:87432 IMAGE:4823475), complete cds	BC066956.1
12	Homo sapiens metallothionein 2A, mRNA (cDNA clone MGC:88284 IMAGE:4051053), complete cds	BC070289.1
13	Homo sapiens N-myc and STAT interactor (NMI), mRNA	NM_004688.3
14	Homo sapiens keratin 18 (KRT18), transcript variant 1, mRNA	NM_000224.3
15	Homo sapiens SIX homeobox 5 (SIX5), mRNA	NM_175875.5
16	Homo sapiens keratin 18 (KRT18), transcript variant 1, mRNA	NM_000224.3
17	Homo sapiens isolate PNG82 haplogroup Q1 mitochondrion, complete genome	MN849858.1
18	Homo sapiens syndecan binding protein (SDCBP), transcript variant 1, mRNA	NM_005625.4
19	Yeast vector pDEST-GADT7, complete sequence	LT727257.1
20	Homo sapiens keratin 18 (KRT18), transcript variant 1, mRNA	NM_000224.3
21	Homo sapiens proteasome 20S subunit beta 10 (PSMB10), mRNA	NM_002801.4
22	Homo sapiens haplogroup L3b1a mitochondrion, complete genome	MT232742.1
23	Homo sapiens N-myc and STAT interactor (NMI), mRNA	NM_004688.3
24	Homo sapiens proteasome 20S subunit alpha 1 (PSMA1), transcript variant 2, mRNA	NM_002786.4
25	Homo sapiens BTB (POZ) domain containing 2, mRNA (cDNA clone IMAGE:5474960), partial cds	BC069199.1
26	Homo sapiens clone HEL-S-39a epididymis secretory sperm binding protein mRNA, partial cds	GQ891316.1
27	Homo sapiens E2F transcription factor 4 (E2F4), mRNA	NM_001950.4
28	Homo sapiens tubulin beta 6 class V (TUBB6), transcript variant 6, mRNA	NM_001303528.2

29	Homo sapiens clone HEL-S-223 epididymis secretory sperm binding protein mRNA, complete cds	GQ891502.1
30	Positive control	
31	Negative control	

Table S2. SDCBP Molecular Function(Gene Ontology) in Fig 3

Aspect	Description	GO-term
Molecular Function	cell adhesion molecule binding	GO:0050839
Molecular Function	ephrin receptor binding	GO:0046875
Molecular Function	frizzled binding	GO:0005109
Molecular Function	growth factor binding	GO:0019838
Molecular Function	identical protein binding	GO:0042802
Molecular Function	interleukin-5 receptor binding	GO:0005137
Molecular Function	neurexin family protein binding	GO:0042043
Molecular Function	protein C-terminus binding	GO:0008022
Molecular Function	protein heterodimerization activity	GO:0046982
Molecular Function	protein N-terminus binding	GO:0047485
Molecular Function	protein-containing complex binding	GO:0044877
Molecular Function	syndecan binding	GO:0045545

Table S3. siRNA SDCBP

siRNA Name	Sequences(5′ to 3′)
siRNA SDCBP 2	AGAAGAAAUACGUGCAAAUTT
	AUUUGCACGUAUUUCUUCUTT
siRNA SDCBP 5	GAACAUAACAUCUGUGAAATT
	UUUCACAGAUGUUAUGUUCTT
siRNA SDCBP 7	GCACCAAGCAUUAUGAAAATT
	UUUUCAUAAUGCUUGGUGCTT

Table S4. qPT-PCR primers used in this study

Primers Name	Sequences(5′to 3′)	TM	Gene ID
SDCBP	F:ACTGTCAACATCTGGGACTGTAGTTAC	57.9	NC_000008.11
	R:GCTTGGTGCCATCCGCTTAATAATATG	58.2	
EFNB1	F:TGGCAAGCATGAGACTGTGAACC	60.2	NC_000023.11
	R:ATGAGCAGGAAGATGACGCAACC	60.0	
IL5RA	F:GCCAAGAATACAGCAAAGACACACTG	58.4	NC_000003.12
	R:TAACAAGCACCGCAAGCCAGTC	60.9	
IL5	F:CTTGGAGCTGCCTACGTGTATGC	60.8	NC_000005.10

	R:GAACAGGAATCCTCAGAGTCTCATTGG	58.9	
NFASC	F:GGAGCCCATCACCCAAGACAAAC	61.2	NC_000001.11
	R:GGCGTTACAACTGTAGTCGGTCTG	60.1	
ANXA2	F:AGCATCAGGAAAGAGGTTAAAGGAGAC	58.2	NC_000015.10
	R:CTTCATGGAGTCATACAGCCGATCAG	59.3	
EFNB2	F:GGTTCTAGCACAGACGGCAACAG	60.9	NC_000013.11
	R:GGACCACCAGCGTGATGATGATG	60.9	
SDC1	F:GGAGACAGCATCAGGGTTAAGAAGAC	59.3	NC_000002.12
	R:GCGACAAACTCAAGAGACAACACAC	58.6	
SDC4	F:TGTCCAACAAGGTGTCAATGTCCAG	59.5	NC_000020.11
	R:CAGTAGGATCAGGAAGACGGCAAAG	59.6	
SDC2	F:GACCCAGCCGAAGAGGATACAAATG	59.9	NC_000008.11
	R:AGCCAATAACTCCACCAGCAATGAC	59.6	
EPHB2	F:AAGGTGCTTTCTGCTTACTGACTTAGG	58.5	NC_000001.11
	R:CAGGTGGGAGGAGGGAAGAGTG	62.4	
GAPDH	F: GGAGCGAGATCCCTCCAAAAT	58.33	
	R: GGCTGTTGTCATACTTCTCATGG	59.25	

Table S5. Antibodies used in this study

Antibodies	Source	Identifier
Anti-6×His Tag rabbit pAb	Abcam, UK	ab9108
Anti-6×His Tag mouse mAb	Abcam, UK	ab18184
Anti-Myc tag mouse mAb	Abcam, UK	ab32
Mouse Anti-β actin mAb	ZSGB-Bio, China	TA-09
Goat Anti-Rabbit IgG H&L (HRP)	Abcam, UK	ab6721
Goat Anti-Mouse IgG H&L (HRP)	Abcam, UK	ab6789
Syntenin-1 Polyclonal Antibody	Proteintech, USA	22399-1-AP
CD138/Syndecan-1 Monoclonal Antibody	Proteintech, USA	60185-1-Ig
SDC2 Monoclonal Antibody	Proteintech, USA	67088-1-Ig
SDC4 Polyclonal Antibody	Proteintech, USA	11820-1-AP
Mouse monoclonal to CD63	Abcam, UK	ab193349
Rabbit monoclonal to CD9	Abcam, UK	ab92726
Mouse monoclonal to Hsp70	Abcam, UK	ab2787

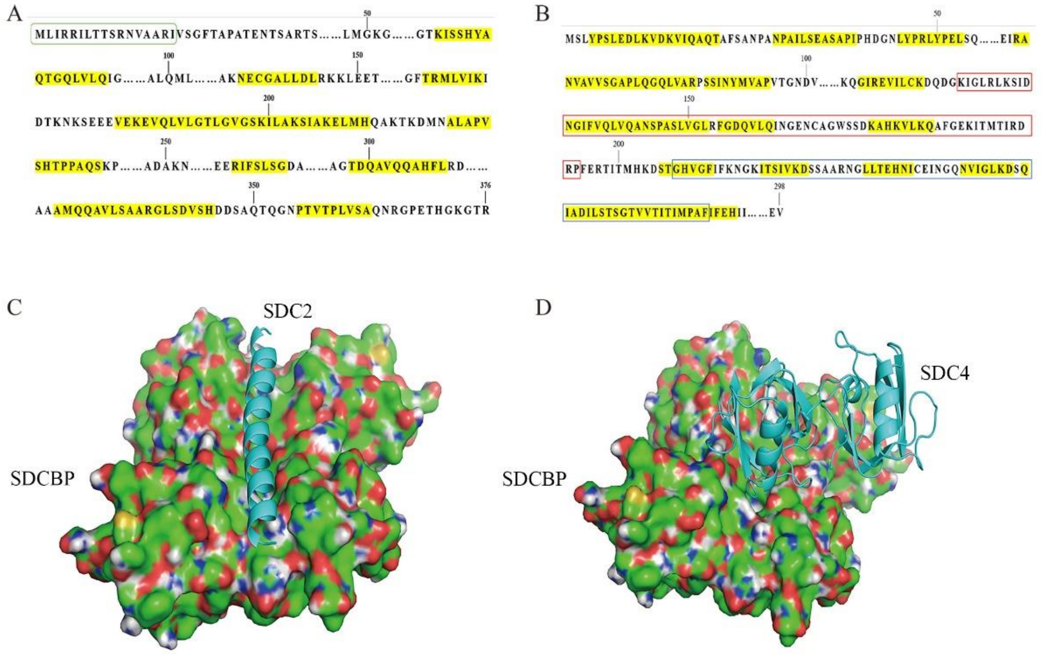

Fig. S1 Bioinformatics analysis for Ats-1 and SDCBP. A. Schematic representation of the structure of Ats-1. Yellow highlights indicate antigenic determinants. Green boxes indicate mitochondrial localization signals. B. Schematic structure of SDCBP. Yellow highlights indicate antigenic determinants. Red and blue boxes indicate PDZ domains. D. Homology models for SDCBP and SDC2 were built by SWISS-MODEL. Protein–protein docking between SDCBP and SDC2 was established with ClusPro 2.0. E. Homology models for SDCBP and SDC4 were built by SWISS-MODEL. Protein–protein docking between SDCBP and SDC4 was established with ClusPro 2.0.

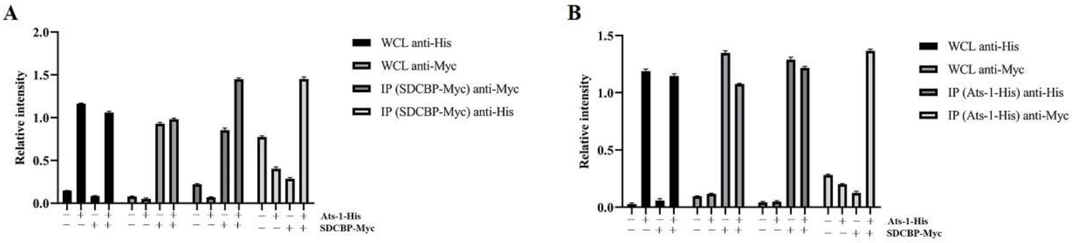

Fig. S2 Interaction between Ats-1 and SDCBP. Plasmids encoding Ats-1-His and SDCBP-Myc were co-transfected into HEK293T cells. After 48 h, cells were collected and the whole-cell lysates were immunoprecipitated with anti-His antibody (left) or anti-Myc antibody (right), and Protein A/G agarose beads. Ats-1 and SDCBP were then detected with western blotting assays using the antibodies against either the His or Myc tags. Quantitative analysis of western blot results was performed using ImageJ software.

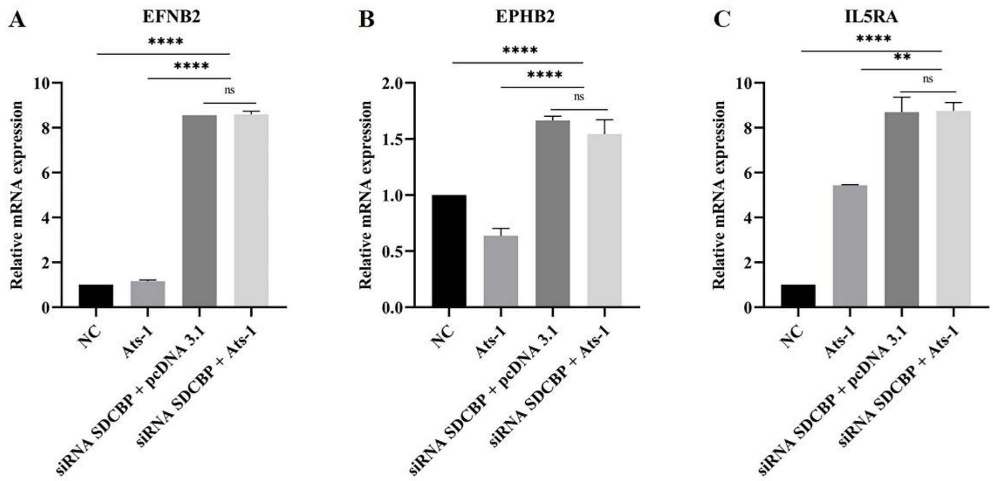

Fig. S3 qPCR of the expression levels of SDCBP related proteins induced by Ats-1. A. mRNA expression level of EFNB2 before and after siRNA SDCBP /Ats-1 transfer. B. mRNA expression level of EPHB2 before and after siRNA SDCBP /Ats-1 transfer. C. mRNA expression level of IL5RA before and after siRNA SDCBP/Ats-1 transfer. All data are shown as mean ± SD from three independent tests. ns, no significant difference, *$p < 0.05$, **$p < 0.01$, ****$p < 0.0001$.

The original and unprocessed blot images of Figure 1.B

HEK293T cells（Ats-1）

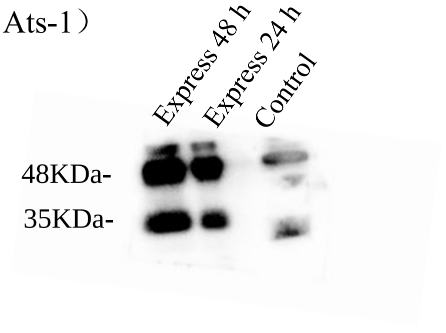

HEK293T cells（β-actin）

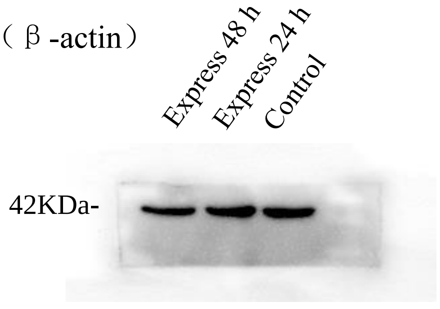

The original and unprocessed blot images of Figure 2.C

IP: His (Ats-1) IB: Myc

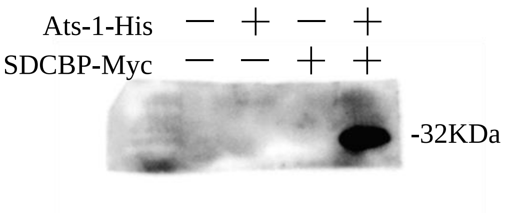

IP: His (Ats-1) IB: His

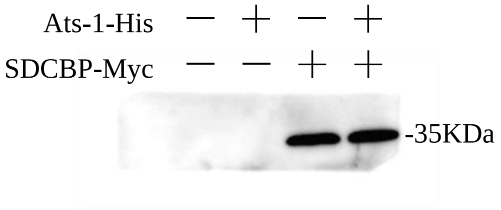

WCL IB: Myc (left)

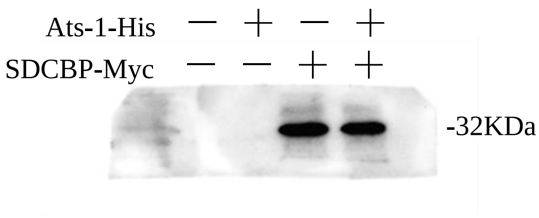

WCL IB: His (left)

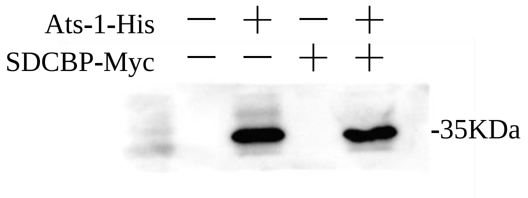

WCL IB: β-actin (left)

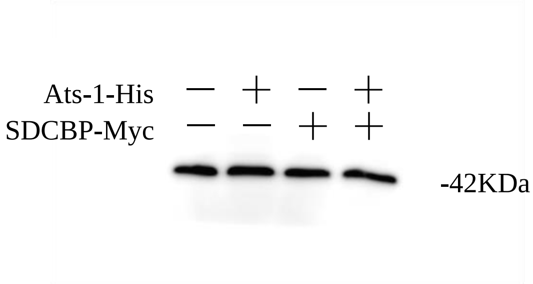

IP: Myc (SDCBP)　IB: His

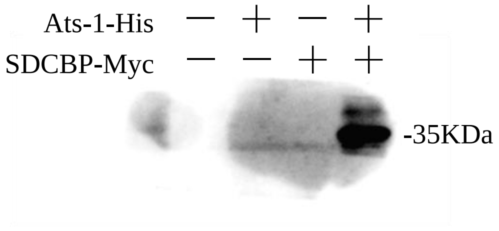

IP: Myc (SDCBP)　IB: Myc

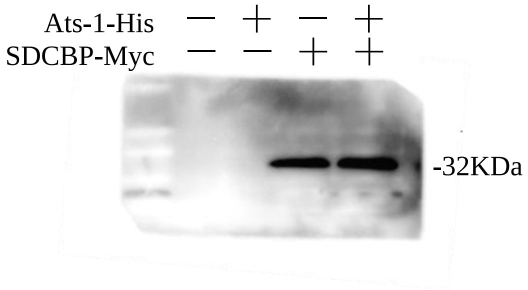

WCL　IB: Myc (right)

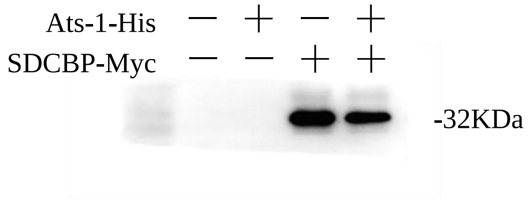

WCL　IB: His (right)

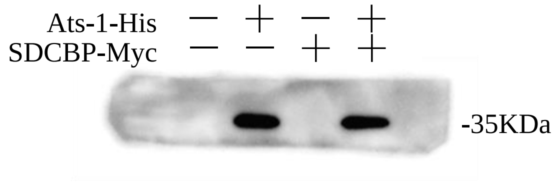

WCL　IB: β-actin (right)

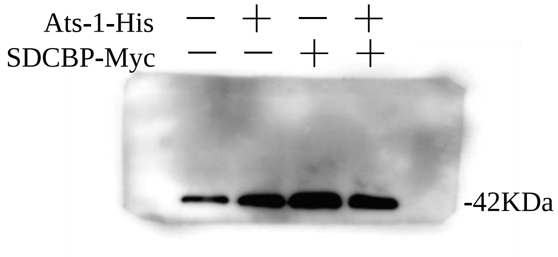

The original and unprocessed blot images of Figure 5.A

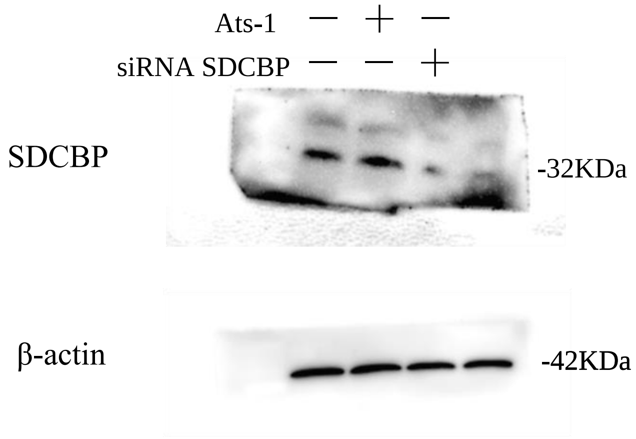

The original and unprocessed blot images of Figure 5.B

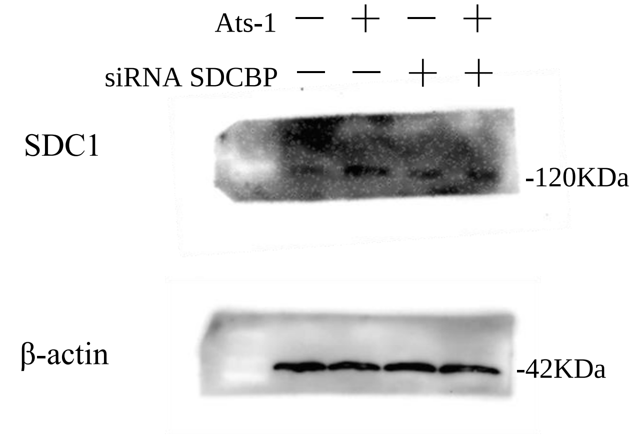

The original and unprocessed blot images of Figure 5.C

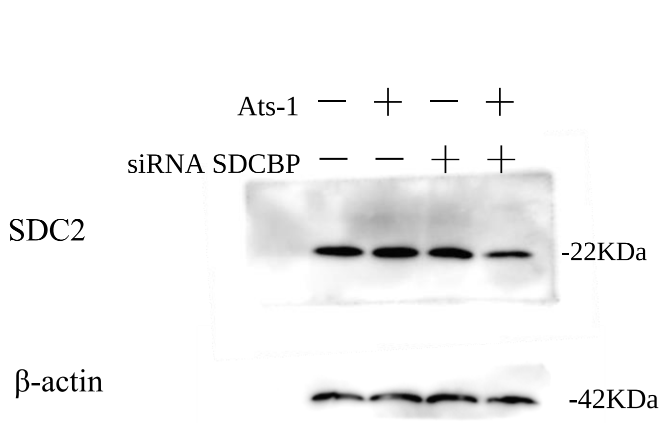

The original and unprocessed blot images of Figure 5.D

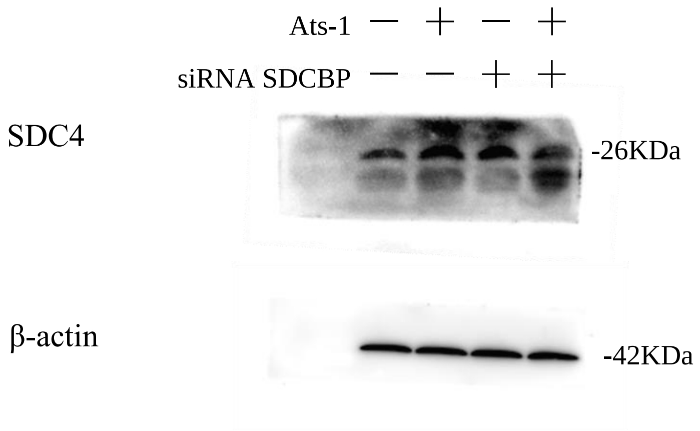

The original and unprocessed blot images of Figure 6.D

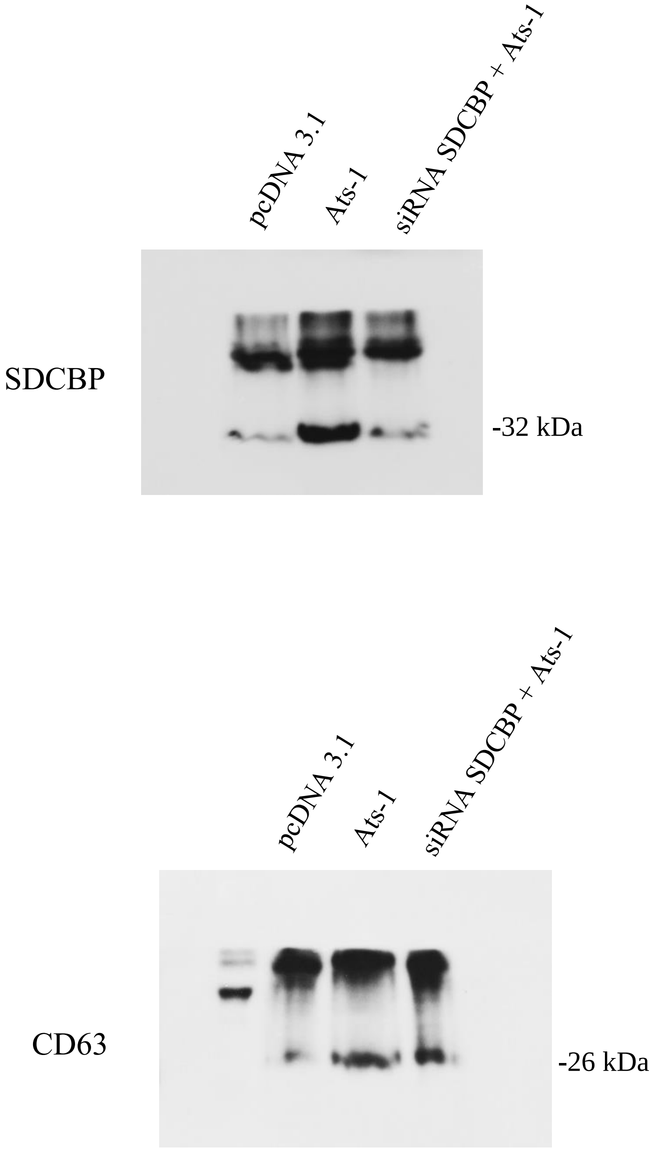

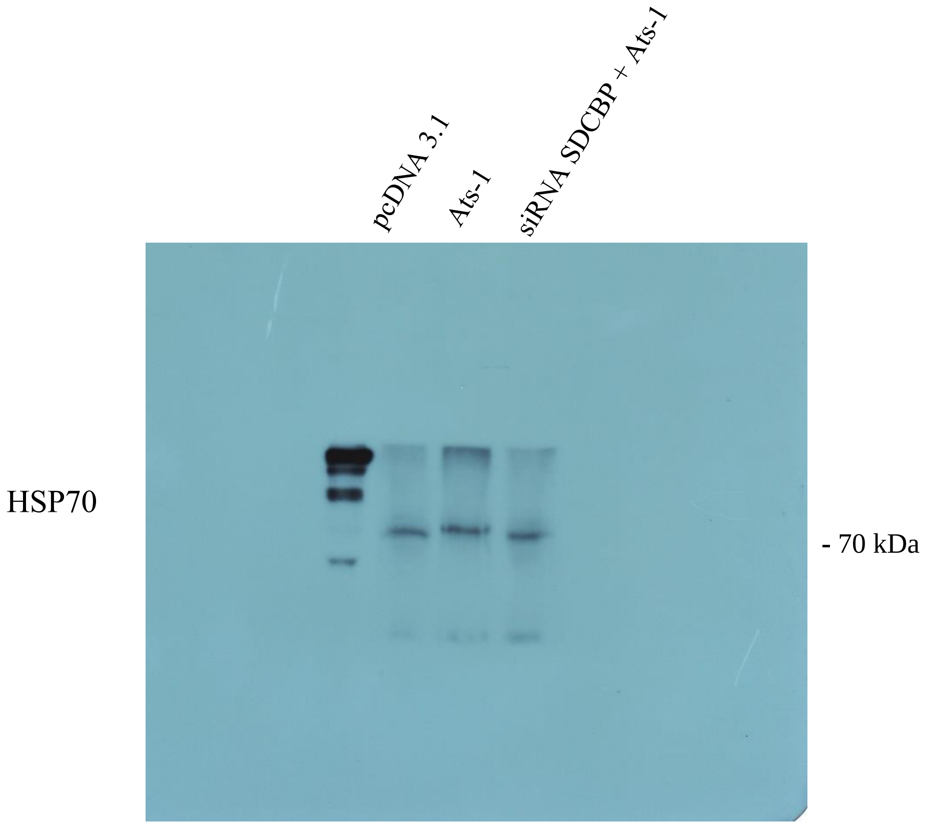
pcDNA 3.1
Ats-1
siRNA SDCBP + Ats-1
HSP70
- 70 kDa

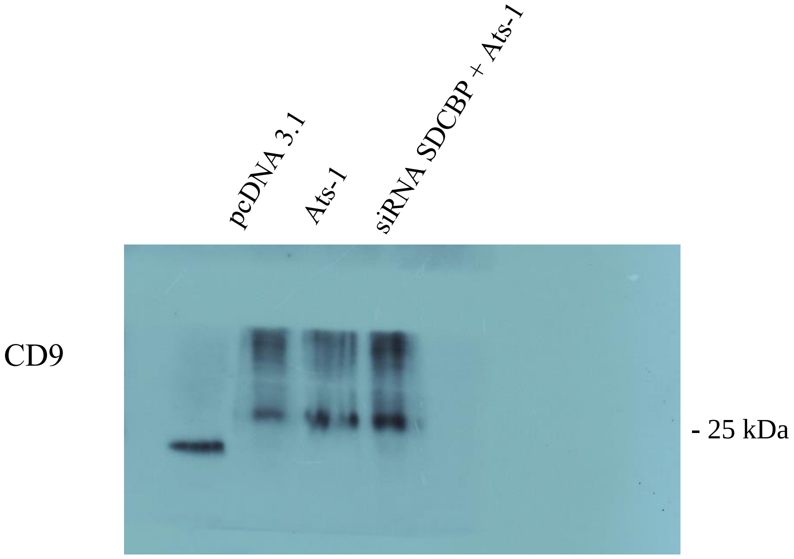
pcDNA 3.1
Ats-1
siRNA SDCBP + Ats-1
CD9
- 25 kDa
